# Intestinal permeability to iohexol as an in vivo marker of chemotherapy-induced gastrointestinal toxicity in Sprague–Dawley rats

**DOI:** 10.1007/s00280-016-3150-3

**Published:** 2016-09-02

**Authors:** Richard A. Forsgård, Riitta Korpela, Reetta Holma, Jere Lindén, Rafael Frias, Thomas Spillmann, Pia Österlund

**Affiliations:** 1 Pharmacology, University of Helsinki, P.O. Box 63, 00014 Helsinki, Finland; 2 Department of Veterinary Biosciences, Faculty of Veterinary Medicine, University of Helsinki, Helsinki, Finland; 3 Central Animal Laboratory, University of Turku, Turku, Finland; 4 Comparative Medicine, Karolinska Institutet, Stockholm, Sweden; 5 Department of Equine and Small Animal Medicine, Faculty of Veterinary Medicine, University of Helsinki, Helsinki, Finland; 6 Department of Oncology, University of Helsinki and Helsinki University Hospital, Helsinki, Finland

**Keywords:** Intestinal permeability, Chemotherapy, 5-Fluorouracil, Oxaliplatin, Irinotecan

## Abstract

**Purpose:**

Gastrointestinal toxicity is the most common adverse effect of chemotherapy. Chemotherapeutic drugs damage the intestinal mucosa and increase intestinal permeability. Intestinal permeability is one of the key markers of gastrointestinal function and measuring intestinal permeability could serve as a useful tool for assessing the severity of chemotherapy-induced gastrointestinal toxicity.

**Methods:**

Male Sprague–Dawley rats were injected intraperitoneally either with 5-fluorouracil (150 mg/kg), oxaliplatin (15 mg/kg) or irinotecan (200 mg/kg). Clinical signs of gastrointestinal toxicity were assessed daily by weighing the animals and by checking for diarrhea. After 48 h, intestinal permeability to iohexol was measured in vivo by giving the animals 1 ml of 647 mg/ml iohexol solution by oral gavage and collecting all the excreted urine for 24 h. All of the animals were euthanized 72 h after drug administration and tissue samples were harvested from the jejunum and colon.

**Results:**

All chemotherapeutics caused significant body weight loss and diarrhea. Intestinal permeability to iohexol was also increased in all treatment groups and histological analysis revealed significant intestinal damage in both jejunum and colon. Iohexol permeability correlated with the severity of clinical signs of gastrointestinal toxicity and with acute colonic injury.

**Conclusions:**

Chemotherapeutic drugs, such as 5-fluorouracil, oxaliplatin, and irinotecan, increase intestinal permeability to iohexol. Measuring intestinal permeability to iohexol could provide a simple marker for assessing chemotherapy-induced gastrointestinal toxicity.

**Electronic supplementary material:**

The online version of this article (doi:10.1007/s00280-016-3150-3) contains supplementary material, which is available to authorized users.

## Introduction

Gastrointestinal (GI) toxicity is the most common adverse effect of chemotherapy and a cause of a variety of symptoms [1, 2]. Symptoms relating to chemotherapy-induced GI toxicity (CIGT) such as diarrhea, vomiting, weight loss, and infections [2] greatly impact patients’ quality of life and may also significantly affect the outcome of the treatment. However, currently no objective methods are available to assess the severity or occurrence of CIGT in individual patients [1].

Chemotherapeutic drugs damage the intestinal mucosa by directly affecting the normal cellular turnover of enterocytes. However, the pathophysiology of CIGT seems to be a multifactorial process that extends beyond simple epithelial damage [3]. Studies have shown that chemotherapy compromises the mucosal barrier function and increases intestinal permeability [4–7]. Intestinal permeability (IP) is one of the key parameters of normal GI function, and multiple diseases have shown an association with alterations in IP [8, 9]. Measuring IP could therefore be a useful tool to objectively assess and predict the severity of CIGT, as well as to follow-up treatment safety.

The primary purpose of this study was to examine whether the severity of CIGT correlates with IP to iohexol. Iohexol is routinely used in medical facilities as a contrast medium and has also recently proved to be a reliable and sensitive marker for IP examinations [10–13]. The advantage of using iohexol as an IP marker against other previously used markers is that iohexol is non-radioactive, non-hygroscopic, not degraded by intestinal microbiota, well tolerated, safe, easily detectable, cost-effective, and readily available in medical facilities [10, 11, 14, 15]. Because previous studies have mainly examined the effects of a combination of different chemotherapeutics on IP, our secondary aim was to clarify how individual drugs affect IP. In addition, to further characterize the effects of chemotherapeutic agents, we examined the intestinal tissues histologically and measured the serum levels of zonulin, an endogenous protein that specifically and reversibly regulates intestinal permeability [16].

## Materials and methods

### Ethical statement

The experiment using animals was approved by the National Animal Experiment Board (ESAVI/114/04.10.07/2015).

### Animals

A total of 48 male Hsd:Sprague–Dawley^®^™ SD^®^™ (SD) rats were obtained from Harlan (Udine, Italy) at the age of 6 weeks. The rats were acclimatized for 18 days before entering the study. They were housed under specific pathogen-free laboratory conditions using artificial lightening with a 12-h light/dark cycle (lights on at 6 am) with room temperature of 22 ± 2 °C and relative humidity of 55 ± 15 %. The animals were kept in open stainless steel cages (59.5 × 38.0 × 20 cm) with solid bottoms and Aspen chips as bedding (Tapvei, Harjumaa, Estonia) in social groups of four rats. The animals were fed a rat chow (2018 Teklad Global 18 % Protein Rodent Diet, Harlan Laboratories, Madison, WI, USA) ad libitum and provided free access to tap water in polycarbonate bottles.

### Experimental protocol

After the 18-day acclimatization time and at the commencement of the study, the rats were 8 weeks old and their average body weight was 283 ± 16 g. The animals were randomly assigned into four experimental groups (Control, 5-fluorouracil, oxaliplatin, and irinotecan, *n* = 12 per group). Baseline intestinal permeability was assessed in vivo (*Measurement of intestinal permeability*) after which a 1-ml blood sample was collected from the tail vein under isoflurane (Vetflurane 1000 mg/g, Virbac, Suffolk, UK) anesthesia. The chemotherapeutic drugs were administered after a 13-day recuperation period, and the rats were euthanized following a 3-day (76-h) observation period. During the observation period, the animals were weighted and evaluated for diarrhea (*Diarrhea assessment*) daily, and intestinal permeability measurement was started 48 h after the drug dosing. For euthanasia, the rats were fully anesthetized using isoflurane and subsequently exsanguinated by cardiac puncture and by severing of the aorta.

### Drug administrations

Rats in the experimental group 5-fluorouracil (5-FU) received a single dose of 150 mg/kg 5-fluorouracil (pH adjusted to between 8.6 and 9.4 with sodium hydroxide) (Accord Healthcare, Middlesex, UK) intraperitoneally [17]. The oxaliplatin group received a single intraperitoneal dose of 15 mg/kg oxaliplatin in a vehicle containing tartaric acid and sodium hydroxide as a buffering system (pH between 4.0 and 7.0) (Hospira UK, Warwickshire, UK) [18]. The Irinotecan group was injected intraperitoneally with a 200 mg/kg dose of irinotecan in sorbitol/lactic acid buffer (45 mg/ml of sorbitol, 0.9 mg/ml lactic acid, pH 3.5) (Hospira UK, Warwickshire, UK) [19]. Immediately prior to irinotecan injection, the rats were given 0.01 mg/kg atropine (Leiras, Espoo, Finland) subcutaneously to reduce the irinotecan-induced cholinergic reaction. The control group received a single intraperitoneal injection of 0.9 % saline solution. All injections were administered under isoflurane anesthesia.

### Diarrhea assessment

After the administration of the drugs, the animals were checked daily for diarrhea. The severity of diarrhea was scored accordingly as 0, no diarrhea; (1) mild diarrhea (staining of anus, moist surface of feces); (2) moderate diarrhea (staining of top of legs and lower abdomen, viscous fecal matter); (3) severe diarrhea (staining over legs and higher abdomen, pasty fecal matter) [20].

### Blood sampling

The blood samples were collected in serum separation tubes (VenoSafe™ Clot Act. (Z), Terumo Europe, Leuven, Belgium) and centrifuged at 1500*g* for 10 min. The separated serum was collected and stored in −80 °C for later analysis.

### Measurement of intestinal permeability

The intestinal permeability was assessed with iohexol (Omnipaque 300™, 647 mg iohexol/ml, GE Healthcare, Oslo, Norway). The rats were weighed and given 1 ml of 647 mg/ml iohexol solution by oral gavage. After administration, the animals were immediately placed in individual metabolic cages for urine collection. After 24 h, the amount of collected urine was measured and stored in -18 °C for later analysis. Samples were discarded if fecal contamination or incomplete urine collection was observed.

### Analysis of iohexol

The urine concentration of iohexol was measured by enzyme-linked immunosorbent assay (ELISA) according to the manufacturer’s instructions (BioPAL Inc., Worcester, MA, USA). The percentage of excreted iohexol was calculated using the following equation:

Iohexol (%) = amount of iohexol excreted in urine after 24 h (mg)/amount of administered iohexol (mg) × 100

### Tissue collection

Following euthanasia, the abdomen was opened and the entire intestine was removed. Tissue samples (1 cm) were taken from the middle section of the jejunum and colon. The samples were opened and flushed free of any intestinal content with cold PBS. For histological analysis, the tissue samples were fixed in 10 % neutral buffered formaldehyde (Sigma-Aldrich, St. Louis, MO, USA) for 24–48 h, embedded in paraffin, cut into at 4-μm thick sections, and stained with hematoxylin–eosin (HE).

### Analysis of serum zonulin

The serum concentration of zonulin was measured by ELISA according to the manufacturer’s instructions (BlueGene, Shanghai, China).

### Histological analysis

Jejunum and colon samples were evaluated and mucosal lesions graded separately. As a basis for grading we employed a system originally developed to diagnose gastrointestinal inflammation-related changes in dogs and cats [21], however, modifying it substantially and applying a four-tier scale for grading: minimal (1), mild (2), moderate (3) and marked (4). In jejunal samples, we assessed five change categories: villous stunting, villous epithelial injury, crypt hyperplasia, crypt epithelial injury, and leukocyte infiltration in lamina propria; in the colon comparable five categories were analyzed: surface epithelial injury, crypt hyperplasia, crypt dilatation and distortion, crypt epithelial injury, and leukocyte infiltration in lamina propria. In addition, separate evaluations were made for Paneth cell injury in the jejunum and crypt loss (atrophy) in the colon. Finally, the histopathological grades of jejunal villous epithelial injury, villous stunting, crypt epithelial injury, and Paneth cell injury were combined and averaged to obtain a general measure (score) of acute jejunal injury; correspondingly, for acute colonic injury, the histopathological grades of surface epithelial injury, crypt epithelial injury, and crypt loss were combined and averaged. Histopathological assessment was done in a partly blinded manner. The reader of the slides (JL) was aware of the experimental design and which animals were in the same group but was unaware of the group identities. Criteria for grading and rationale for the selection of the change categories employed in scoring of the acute jejunal and colonic histological injuries are detailed in supplementary data (Online Resource 1).

### Data analysis

Normality of the datasets was tested using Kolmogorov–Smirnov test. Based on this analysis, differences in iohexol permeability, body weight change, diarrhea scores, and histological scores were analyzed using Kruskal–Wallis test and if global *p* < 0.05, Mann–Whitney *U* test was used to calculate the statistical differences between the groups. The results are expressed as medians ± interquartile range. All correlations between variables were calculated as Spearman’s rho correlation coefficients. Statistical calculations were made by PASW Statistics software version 18.0.2 (IBM, Armonk, NY, USA). GraphPad Prism 5 (GraphPad Software Incorporated, La Jolla, CA, USA) was used to create the figures. Histological images were obtained with Axio Imager. A2 microscope (Carl Zeiss, Goettingen, Germany) using a 20× objective. Data were deemed significant when *p* < 0.05.

## Results

### Drug response

**Fig. 1:**
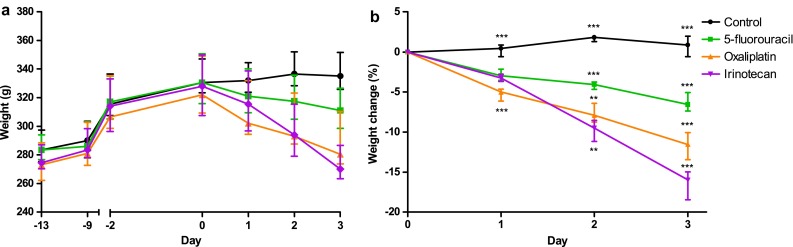
Effects of 5-fluorouracil, oxaliplatin, and irinotecan on the animals’ body weight. **a** Median body weights (g) in different treatment groups from the beginning of the study (Day -13) to the end (Day 3). The drugs were administered on Day 0. **b** All the studied chemotherapeutics caused significant body weight (%) loss already in 24 h compared to the Control group (*n* = 12). Irinotecan (*n* = 12) caused a significantly more severe loss in body weight than oxaliplatin (*n* = 10) and 5-fluorouracil (*n* = 12) in 72 h. However, 5-fluorouracil induced a significantly milder body weight loss than oxaliplatin. *Line graphs* show median with interquartile range. (***p < 0.001 compared to all other groups; **p < 0.01 between groups)

**Fig. 2:**
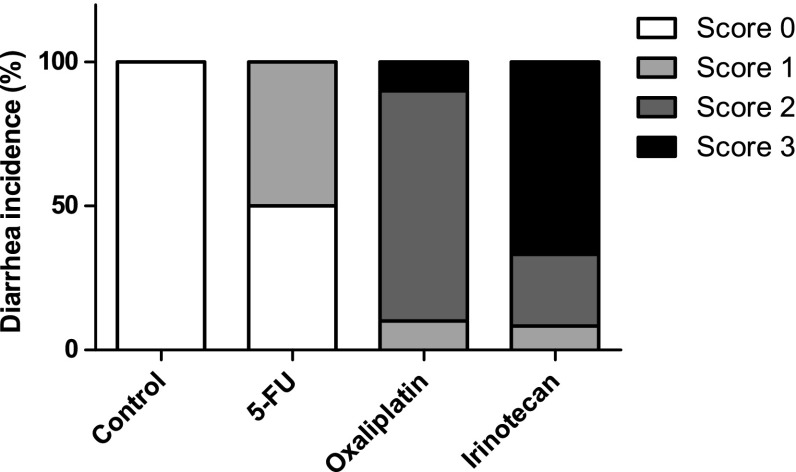
Incidence of diarrhea (%) in different treatment groups 72 h after drug administration. Score 0 = no diarrhea; Score 1 = mild diarrhea (staining of anus, moist surface of feces); Score 2 = moderate diarrhea (staining of top of legs and lower abdomen, viscous fecal matter); Score 3 = severe diarrhea (staining over legs and higher abdomen, pasty fecal matter) [20]. (*n* = 12 in all groups, except in oxaliplatin where *n* = 10)

All drugs caused significant body weight loss compared to the Control group (*p* < 0.001) (Fig. 1a, b). The irinotecan group lost 16.0 ± 3.5 % of their body weight during the experiment (72 h), which was significantly (*p* < 0.001) more than the rats in groups oxaliplatin (11.6 ± 3.4 %) and 5-FU (6.6 ± 2.3 %) (Table 1). There were no differences in body weight between the groups at the start of experiment. In the oxaliplatin group, two rats failed to show any response to the drug and showed similar characteristics in every category as the animals in the Control group. This is most likely due to some problem in the drug administration and they were thus removed from all data analyses. During the experiment, 50 % (6/12) of the 5-FU group developed mild diarrhea. In the oxaliplatin group, 10 % (1/10) had mild diarrhea, 80 % (8/10) moderate, and 10 % (1/10) severe diarrhea. In the irinotecan group, 8 % (1/12) developed mild diarrhea, 25 % (3/12) moderate, and 67 % (8/12) severe diarrhea. No diarrhea was observed in the control group (Fig. 2).

**Table 1:** Intestinal permeability to iohexol (% of administered iohexol) and body weight change in different treatment groups 72 h after drug administration

Group	Iohexol permeability (%)	Δ Body weight (%)
Control	0.47 ± 0.18^b^	0.88 ± 2.6^b^
5-Fluorouracil	1.55 ± 1.46^b,a^	−6.6 ± 2.3^b^
Oxaliplatin	2.61 ± 1.45^b,a^	−11.6 ± 3.4^b^
Irinotecan	8.07 ± 8.90^b^	−16.0 ± 3.5^b^
Kruskal–Wallis	*p* < 0.001	*p* < 0.001

Results are expressed as medians ± interquartile ranges

^a^Statistically significant difference between groups (*p* < 0.05)

^b^Statistically significant difference between groups (*p* < 0.001)

### Permeability to iohexol

**Fig. 3:**
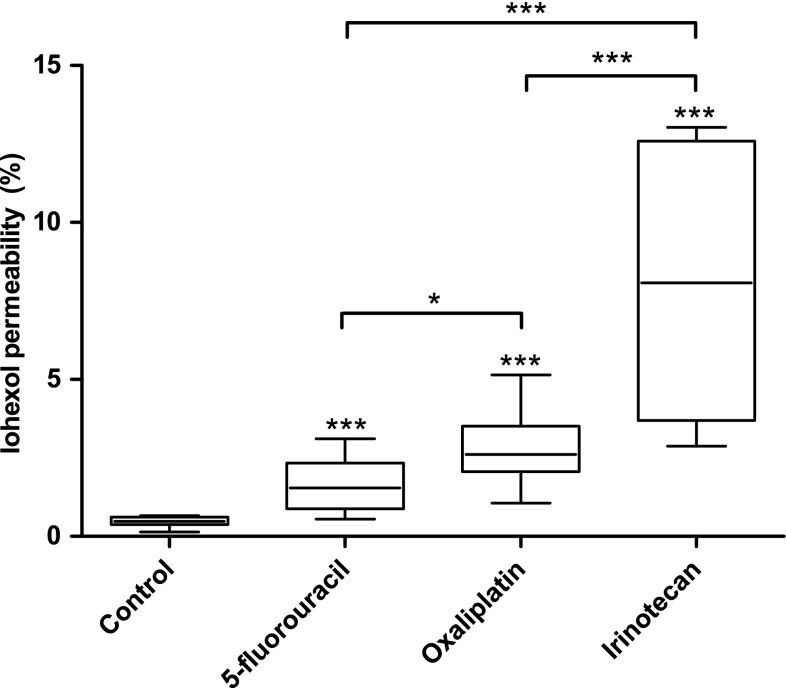
Intestinal permeability to iohexol (% of administered iohexol) in different treatment groups 72 h after drug administration. All the studied chemotherapeutics significantly increased iohexol permeability compared to the Control group (*n* = 12). Irinotecan (*n* = 9) significantly increased iohexol permeability compared to oxaliplatin (*n* = 10) and 5-fluorouracil (*n* = 11). Iohexol permeability was also significantly lower in the 5-fluorouracil group compared to the oxaliplatin group. *Box plots* show median with upper and lower quartiles. Whiskers show minimum and maximum. (*p < 0.05; ***p < 0.001)

Iohexol permeability was significantly (*p* < 0.001) lower in the Control group (0.47 ± 0.18 %) than in the treatment groups. Rats in the 5-FU group (1.55 ± 1.46 %) exhibited significantly (*p* < 0.05) lower iohexol permeability compared to the rats in the oxaliplatin group (2.61 ± 1.45 %). Iohexol permeability was significantly (*p* < 0.001) increased in the irinotecan group (8.07 % ± 8.90) compared to the other groups (Fig. 3; Table 1). A total of four samples were discarded because of fecal contamination: one from the 5-FU group and three from the irinotecan group. There were no differences in the baseline intestinal permeability between the groups (data not shown).

### Correlations between diarrhea scores, body weight change, and permeability to iohexol

**Fig. 4:**
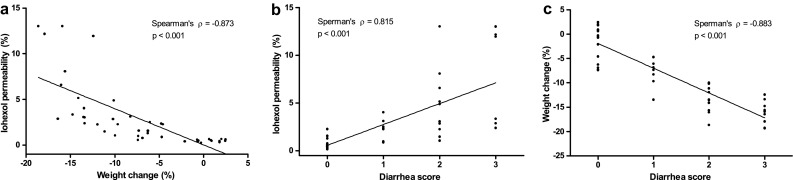
Spearman’s correlations between body weight change and iohexol permeability (**a**), iohexol permeability and diarrhea score (**b**), and body weight change and diarrhea score (**c**). Iohexol permeability correlated inversely with body weight change (Spearman’s rho = −0.873, *p* < 0.001) and positively with diarrhea score (Spearman’s rho = 0.815, *p* < 0.001). There was also a significant inverse correlation between body weight change and diarrhea score (Spearman’s rho = −0.883, *p* < 0.001)

There was an inverse correlation between body weight change and permeability to iohexol (Spearman’s rho: −0.873, *p* < 0.001) (Fig. 4a). Diarrhea scores correlated positively with iohexol permeability (Spearman’s rho: 0.815, *p* < 0.001) (Fig. 4b) and inversely with body weight change (Spearman’s rho: −0.883, *p* < 0.001) (Fig. 4c).

### Serum zonulin

No differences were observed in serum zonulin concentrations between groups (data not shown).

### Histological analysis

**Fig. 5:**
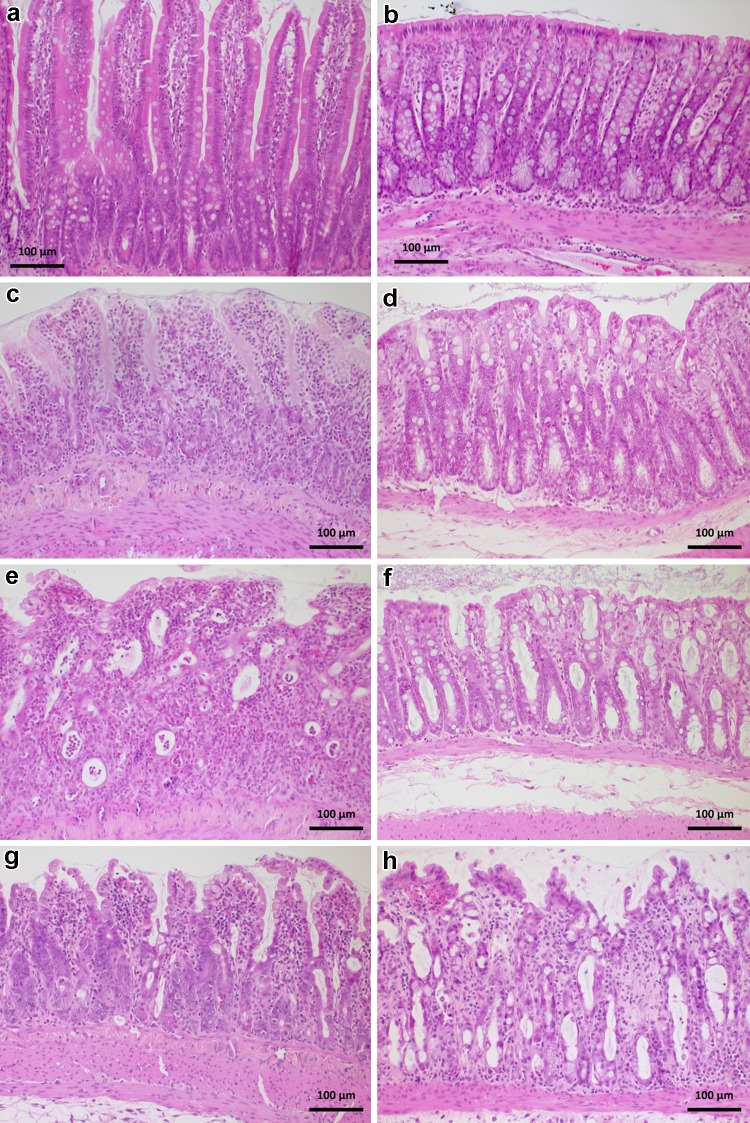
Representative images of the histological findings of jejunum (*left panel*) and colon (*right panel*) in each group: control (**a**, **b**), 5-FU (**c**, **d**), oxaliplatin (**e**, **f**), and irinotecan (**g**, **h**). Jejunal samples of the 5-FU (**c**) and irinotecan (**g**) groups exhibit moderate damage with fused and shortened villi and crypt distortion. In the oxaliplatin group, the jejunal (**e**) surface epithelium and crypts are markedly damaged; surface epithelium lost and crypts collapsed. In the colon, the 5-FU (**d**) and oxaliplatin (**f**) groups display minimal to mild changes. In the Irinotecan group, the surface epithelium of the colon (**h**) is mildly to moderately injured while the crypts are moderately damaged and partly lost

**Fig. 6:**
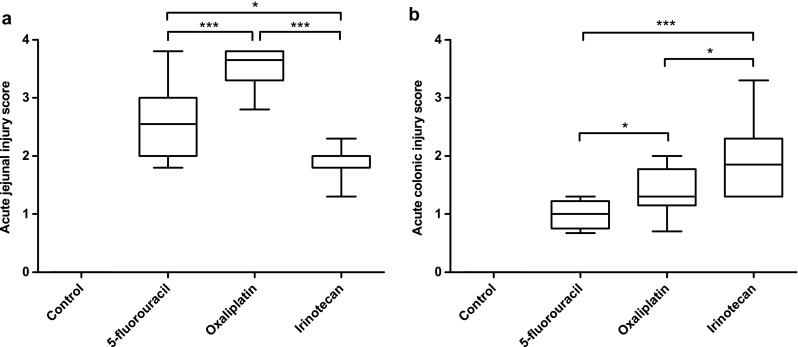
Calculated scores for acute jejunal (**a**) and colonic (**b**) injury. Oxaliplatin (*n* = 10) caused significantly more severe acute injury in the jejunum than 5-fluorouracil (*n* = 12) and irinotecan (*n* = 12). Irinotecan-induced significantly milder damage in the jejunum than 5-fluorouracil. However, in the colon, irinotecan caused the most severe damage. Colonic injury was also more prominent in the Oxaliplatin group than in the 5-fluorouracil group. *Box plots* show median with upper and lower quartiles. *Whiskers* show minimum and maximum. (*p < 0.05; ***p < 0.001)

After parenteral application, all chemotherapeutics caused significant histological changes in the jejunum (Table 2) and colon (Table 3); the changes are summarized in Table 4 and typical alterations depicted in Fig. 5. In the jejunum, the height of the villi was significantly decreased compared to controls (*p* < 0.001). Moderate-to-marked villous epithelial injury was observed in the oxaliplatin group, whereas irinotecan and 5-FU caused mainly mild epithelial damage. Oxaliplatin also caused significantly more severe epithelial injury in the crypts than irinotecan and 5-FU (*p* < 0.001 and *p* = 0.002, respectively). Mild-to-moderate crypt hyperplasia was observed in the irinotecan group which was significantly more prominent compared to oxaliplatin and 5-FU groups (*p* = 0.015 and *p* = 0.001, respectively). Oxaliplatin caused a significant increase in lamina propria leukocytes compared to irinotecan and 5-FU (both *p* < 0.001). Lamina propria leukocytes were also present in the Irinotecan and 5-FU groups although the increase caused by irinotecan was significantly milder compared to 5-FU (*p* = 0.003). Oxaliplatin and 5-FU caused mild-to-moderate Paneth cell injury which was significantly (*p* < 0.001) different from irinotecan that did not cause any observable damage to Paneth cells. The histological score for acute jejunal injury was significantly (*p* < 0.001) larger in the oxaliplatin group (3.7 ± 0.5) than in the other groups (Fig. 5a). Acute jejunal injury was also significantly (*p* < 0.05) more evident in the 5-FU group (2.6 ± 1.0) than in the irinotecan group (2.0 ± 0.2) (Fig. 6a).

**Table 2:** Histopathological grades of the analyzed change categories in the jejunum

Group	Villous stunting	Villous epithelial injury	Crypt hyperplasia	Crypt injury	Paneth cell injury	Lamina propria Leukocytes
*Control*						
5-Fluorouracil	2.8 ± 1.1^a^	2.3 ± 1.3^a^	0.75 ± 1.1^b^	2.9 ± 0.79^a^	2.3 ± 0.49^a,c^	2.9 ± 0.51^b,c^
Oxaliplatin	3.6 ± 0.52^a,b^	3.5 ± 0.70^a,b^	1.5 ± 1.1^a^	4.0 ± 0.0^a,c^	2.8 ± 0.42^a,c^	4.0 ± 0.0^c^
Irinotecan	2.7 ± 0.65^b^	2.3 ± 0.45^b^	2.5 ± 0.52^a,b^	2.7 ± 0.49^c^	0^c^	1.6 ± 1.2^b,c^
Kruskal–Wallis	*p* < 0.001	*p* < 0.001	*p* < 0.001	*p* < 0.001	*p* < 0.001	*p* < 0.001

Results are expressed as means ± standard deviations. 1 = minimal, 2 = mild, 3 = moderate, 4 = marked

^a^Statistically significant difference between groups (*p* < 0.05)

^b^Statistically significant difference between groups (*p* < 0.01)

^c^Statistically significant difference between groups (*p* < 0.001)

**Table 3:** Histopathological grades of the analyzed change categories in the colon

Group	Surface epithelial injury	Crypt hyperplasia	Crypt dilatation and distortion	Crypt injury	Crypt atrophy	Lamina propria Leukocytes
*Control*						
5-Fluorouracil	1.3 ± 0.62^a,c^	1.7 ± 0.78	2.3 ± 0.65	1.8 ± 0.45^b^	0^b^	0.33 ± 0.78^c^
Oxaliplatin	1.9 ± 0.87^a^	2.0 ± 0.0^a^	1.8 ± 0.63	2.2 ± 0.63	0^b^	0.80 ± 1.0^a^
Irinotecan	2.3 ± 0.49^c^	1.0 ± 1.0^a^	2.2 ± 0.39	2.7 ± 0.65^b^	0.83 ± 1.0^b^	1.9 ± 0.67^a,c^
Kruskal–Wallis	*p* < 0.001	*p* < 0.001	*p* < 0.001	*p* < 0.001	*p* < 0.001	*p* < 0.001

Results are expressed as means ± standard deviations. 1 = minimal, 2 = mild, 3 = moderate, 4 = marked

^a^Statistically significant difference between groups (*p* < 0.05)

^b^Statistically significant difference between groups (*p* < 0.01)

^c^Statistically significant difference between groups (*p* < 0.001)

**Table 4:** Summary of the histopathological changes

Group	Jejunum	Colon
5-Fluorouracil	Moderate villous stunting Mild epithelial injury Minimal crypt hyperplasia Moderate crypt injury Mild-to-moderate Paneth cell injury Moderate infiltration of LCs to lamina propria	Minimal surface epithelial injury Mild crypt hyperplasia Mild crypt dilatation and distortion Mild crypt injury No observable crypt atrophy No to minimal infiltration of LCs to lamina propria
Oxaliplatin	Marked villous stunting Moderate-to-smarked epithelial injury Mild crypt hyperplasia Marked crypt injury Mild-to-moderate Paneth cell injury Marked infiltration of LCs to lamina propria	Mild-to-moderate surface epithelial injury Mild crypt hyperplasia Mild crypt dilatation and distortion Mild crypt injury No observable crypt atrophy Minimal infiltration of LCs to lamina propria
Irinotecan	Moderate villous stunting Mild epithelial injury Mild-to-moderate crypt hyperplasia Moderate crypt injury No Paneth cell injury Mild infiltration of LCs to lamina propria	Mild-to-moderate surface epithelial injury Mild crypt hyperplasia Mild crypt dilatation and distortion Moderate crypt injury Minimal crypt atrophy Mild infiltration of LCs to lamina propria

*LCs* leukocytes

In the colon, mild-to-moderate surface epithelial injury was observed in the irinotecan and oxaliplatin groups and the degrees of injury were significantly larger than in the 5-FU group (*p* = 0.001 and *p* = 0.011, respectively). Irinotecan caused also significantly more damage in the crypt epithelium than 5-FU (*p* = 0.001). Mild crypt hyperplasia was observed in all of the treatment groups. Irinotecan increased lamina propria leukocytes significantly more than oxaliplatin and 5-FU (*p* = 0.010 and *p* < 0.001, respectively). Crypt atrophy was only observed in the irinotecan group. The histological score for acute colonic injury was significantly higher in the irinotecan group (1.9 ± 1.0) than in the oxaliplatin group (1.4 ± 0.6, *p* < 0.05) and in the 5-FU group (1.0 ± 0.5, *p* < 0.001) (Fig. 6b). Acute colonic injury was also significantly (*p* = 0.018) more prominent in the oxaliplatin group than in the 5-FU group (Fig. 5b).

### Correlations between permeability to iohexol, histological scores, and body weight change

**Fig. 7:**
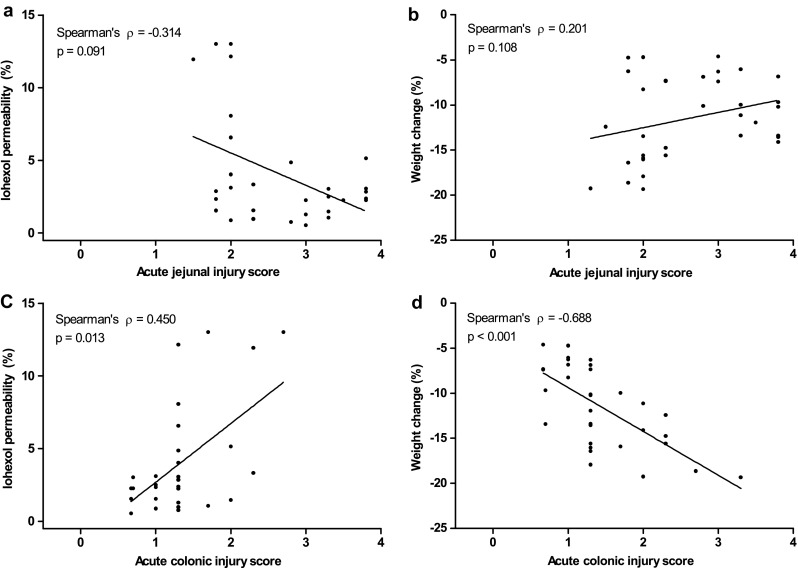
Spearman’s correlations between iohexol permeability and acute jejunal injury score (**a**), body weight change and acute jejunal injury score (**b**), iohexol permeability and acute colonic injury score (**c**), and body weight change and acute colonic injury score (**d**). Acute colonic injury scores correlated positively with iohexol permeability (Spearman’s rho = 0.450, *p* < 0.013) and inversely with body weight change (Spearman’s rho = −0.688, *p* < 0.001). No significant correlations were observed between acute jejunal injury score and iohexol permeability (*p* = 0.091) and between acute jejunal injury score and body weight change (*p* = 0.108)

Iohexol permeability and histological score for acute colonic injury (Fig. 7c) showed a significant positive correlation (Spearman’s rho = 0.450, *p* = 0.013). Conversely, a significant inverse correlation was observed between acute colonic injury and body weight change (Spearman’s rho = -0.688, *p* < 0.001) (Fig. 7d). Acute jejunal injury showed no significant correlation either with iohexol permeability or with body weight change (Fig. 7a, b). The control group was omitted from this correlation analysis because their histological scores were all zero and thus they skewed the correlation toward the control values.

## Discussion

The aim of our study was to investigate whether intestinal permeability to iohexol accurately reflects the severity of chemotherapy-induced gut toxicity. Previous studies have shown that chemotherapeutic agents can increase intestinal permeability [4, 22–24], but the effects of individual agents remain relatively unknown.

Results from our study show that 5-FU, oxaliplatin, and irinotecan can all damage the intestinal mucosa and increase intestinal permeability to iohexol. In addition, iohexol permeability reflected the severity of gut toxicity observed as body weight loss, diarrhea symptoms, and histological injury in the colon. These interesting findings raise the question whether iohexol permeability could be used as a marker of CIGT in the clinical practice. Halme et al. [13] have previously concluded that intestinal permeability to iohexol is an accurate marker of disease activity in patients with inflammatory bowel disease (IBD). Because IBDs and CIGT share similar clinical features, iohexol permeability could also serve as a tool to objectively assess CIGT in patients. Some evidence already exists that increased intestinal permeability could correlate with the severity of CIGT [5–7, 22, 25]. For example, Russo et al. [5] found that a chemotherapy regimen consisting of 5-FU, epirubicin, and cyclophosphamide increased intestinal permeability (measured as lactulose/mannitol ratio) significantly more in patients who were suffering from chemotherapy-induced diarrhea compared with patients who did not develop diarrhea. Melichar et al. [7] studied the effects of paclitaxel and platinum on intestinal barrier function and found that those patients that developed the most severe diarrhea had also the largest increase in intestinal permeability to different sugar probes. Interestingly, they also found that pre-chemotherapy intestinal permeability values were elevated in patients with the most severe diarrhea grades [7]. This indicates that measuring intestinal permeability before chemotherapy could possibly identify those patients that are the most susceptible to CIGT and thus allow clinicians adjust their dosing accordingly. However, other studies have not found any differences in pre-chemotherapy intestinal permeability values and the risk of severe CIGT [5, 23]. Thus, the association between the risk of CIGT and intestinal permeability requires more research.

The exact molecular mechanisms by which chemotherapeutic agents cause gut toxicity remain unknown. Recent preclinical studies have focused on the role of tight junctions in CIGT. Tight junctions are protein complexes that connect adjacent epithelial cells together and limit solute flux through the paracellular space [26]. Solutes can cross the epithelial barrier either paracellularly by passive diffusion through tight junctions or transcellularly by crossing through the cell membranes. Usually, the paracellular pathway is more permeable than the transcellular route making tight junctions key regulators of intestinal permeability [26]. Intestinal permeability is also regulated by zonulin. Zonulin is a physiological regulator of tight junctions and high serum concentrations of zonulin have been associated with increased intestinal permeability [27]. Previously, Russo et al. [5] studied the serum zonulin concentrations of patients receiving chemotherapy and did not observe any changes during the treatment even though they observed an increase in intestinal permeability. Similarly, we also did not observe any differences in zonulin concentrations between the groups despite the increased intestinal permeability to iohexol. Because iohexol permeates the epithelia passively through the tight junctions [15], these findings indicate that chemotherapeutics affect tight junctions through mechanisms that are not zonulin-dependent. Studies have shown that chemotherapeutics can affect the expression of tight junctional proteins. Wardill et al. [28] showed that irinotecan decreases the expression of tight junctional proteins in the small and large intestine. However, they did not examine whether this change in protein expression increases intestinal permeability. Nakao et al. [29] observed a similar irinotecan-induced decrease in tight junctional protein expression in the intestine. They were also able to show that irinotecan treatment decreases transepithelial resistance in the colon suggesting increased intestinal permeability to macromolecules [29]. These findings together with our results imply that the irinotecan-induced gut toxicity is at least in part mediated by tight junctional damage leading to increased intestinal permeability. This can also be the case regarding 5-FU and oxaliplatin although little is known about their effects on tight junctional proteins. 5-FU increases intestinal permeability to technetium-labeled diethylene-triamine-pentaacetate (Tc-99 m-DTPA) in mice [30, 31] and to chromium-labeled ethylenediaminetetraacetate (^51^Cr-EDTA) in humans [6, 25] but the role of tight junctional protein expression in this process remains uncharacterized. The data regarding the effects of oxaliplatin are even scarcer and to our knowledge this is the first study to show that oxaliplatin causes a significant increase in intestinal permeability. However, more research is needed to elucidate the role of tight junctions behind these changes.

The histopathological findings in our study show significant mucosal damage expressing as villous stunting and crypt destruction. These results are in line with previous studies [18, 28, 32]. Overall, the histopathological alterations were more pronounced in the small intestine than in the colon, although the grading between the two sites is not directly comparable. However, intestinal permeability to iohexol and the change in body weight correlated with the degree of acute colonic injury. This suggests that, in addition to the clinical signs of CIGT, increased intestinal permeability to iohexol can also reflect the extent of chemotherapy-induced pathological changes in the colon. This was not the case in the small intestine where iohexol permeability did not correlate with the degree of acute intestinal injury in the jejunum. This could be due to our study setting where we collected all the excreted urine in 24 h and not multiple samples during the day. Therefore, we cannot distinguish between small intestinal and colonic iohexol permeability and may only see the difference in the permeability of the whole intestine. Notably, in human IBD iohexol permeability was highest in patients with moderate-to-severe disease activity in the colon [13]. Our histopathological findings also reveal that there seems to be some differences in the mechanism of toxicity between the studied chemotherapeutics. For example, oxaliplatin caused more severe damage in the small intestine than 5-FU and irinotecan. Irinotecan on the other hand affected the large intestine more severely than oxaliplatin and 5-FU, even resulting to minimal mucosal atrophy (loss of crypts) in the colon. The irinotecan-induced colonic damage is probably due to microbial β-glucuronidase that converts the glucuronidated irinotecan metabolite 7-ethyl-10-hydroxycamptothecin (SN-38G) back to its toxic form SN-38 [33]. SN-38 is the active metabolite of irinotecan that is largely responsible for its toxicity. This added toxicity of irinotecan could also explain why we observed the most severe clinical signs of CIGT and increased intestinal permeability in the irinotecan group. There were also some differences in crypt damage between the groups. We killed all the animals after 72 h, and at this time point jejunal crypt damage was the most severe in the oxaliplatin group. However, at the same time the irinotecan group expressed extensive crypt hyperplasia. Crypt hyperplasia is usually a follow-up reaction to crypt damage which indicates that the most severe crypt damage happened earlier in the irinotecan group. This is supported by the findings of Wardill et al. [28] who reported the most severe irinotecan-induced jejunal crypt damage between 48 and 72 h. Interestingly, 5-FU and oxaliplatin caused significantly more leukocyte infiltration in the jejunal lamina propria than irinotecan. This effect was reversed in the colon where irinotecan-induced leukocyte infiltration in the lamina propria. These findings highlight the role of inflammatory reaction in CIGT. Reports have shown that proinflammatory cytokines contribute to the pathophysiology of CIGT [34–36] and researchers have had some success alleviating CIGT with immunosuppressive substances [18, 37, 38]. Given that proinflammatory cytokines can disrupt the mucosal barrier function and increase intestinal permeability [39], the interplay between the two is probably one of the key factors in the pathophysiology of CIGT.

In conclusion, our results show that 5-FU, oxaliplatin, and irinotecan increase in vivo intestinal permeability to iohexol. Iohexol permeability correlated with clinical manifestations of CIGT such as diarrhea severity and body weight loss, and with histopathological scoring for acute injury in the colon. Thus, measuring iohexol permeability shows promise to potentially provide a simple marker for objectively assessing the severity of CIGT and helping to understand the relationship between the pathophysiology of CIGT and intestinal permeability. However, further research is needed to refine the methodology and validate its usefulness for clinical applications.

## Electronic supplementary material

Below is the link to the electronic supplementary material.

Supplementary material 1 (PDF 659 kb)
