# Supplementary material for: Intestinal permeability to iohexol as an in vivo marker of chemotherapy-induced gastrointestinal toxicity in Sprague–Dawley rats
Source: Cancer Chemother Pharmacol. 2016 Sep 2;78(4):863–74. doi: 10.1007/s00280-016-3150-3 (PMC5040744; doi:10.1007/s00280-016-3150-3)
Supplement: Supplementary file 1 — Supplementary material 1 (PDF 659 kb) [file 280_2016_3150_MOESM1_ESM.pdf]

Supplementary data 1

INTESTINAL PERMEABILITY TO IOHEXOL AS AN *IN VIVO* MARKER OF CHEMOTHERAPY-INDUCED GASTROINTESTINAL TOXICITY IN SPRAGUE-DAWLEY RATS

Richard A. Forsgård[1], *Riitta Korpela*[1], *Reetta Holma*[1], *Jere Lindén*[2], *Rafael Frias*[3,4], *Thomas Spillmann*[5], *Pia Österlund*[6]

[1]University of Helsinki, Faculty of Medicine, Pharmacology, Finland

[2]Department of Veterinary Biosciences, Faculty of Veterinary Medicine, University of Helsinki, Finland

[3]Central Animal Laboratory, University of Turku, Finland

[4]Comparative Medicine, Karolinska Institutet, Stockholm, Sweden

[5]Department of Equine and Small Animal Medicine, Faculty of Veterinary Medicine, University of Helsinki, Finland

[6]Department of Oncology, University of Helsinki and Helsinki University Hospital, Finland

Corresponding author: Richard Forsgård, Faculty of Medicine, Pharmacology, P.O. Box 63, FI-00014 University of Helsinki, Finland, richard.forsgard@helsinki.fi Telephone: +358-50-448-2227 Fax: +358-9-4546168

GRADING OF MUCOSAL CHANGES

The mucosal changes were subjectively graded employing a four-tier system; minimal, mild, moderate or marked, and then assigning a numerical value 1, 2, 3 or 4 to each respective grade. In tables 2 and 3 lesion stages per treatment was illustrated by averages and standard deviations, while comparisons were done applying nonparametric statistical methods. In some changes the first grade (minimal; 1) was not applicable and the grading was thus started from the second grade (mild; 2). Paneth cell injury was assessed using two tiers.

Inflammatory activity in the lamina propria (lamina propria leucocytes) was graded by first assessing eosinophilic granulocyte, neutrophilic granulocyte, and lymphocyte numbers individually; and then, if the infiltrate consisted of more than one type of inflammatory cells, summing the numerical values together. This resulted to a “final” single inflammatory grade, the maximum numerical value of which was, however, limited to 4 (marked). Individual leucocytes were graded employing three tiers; mild, moderate or marked.

Crypt hyperplasia was generally lenient and we applied somewhat different grading system for jejunum and colon samples due to related villous stunting assessment in jejunum and evaluation of (epithelial injury –related) crypt dilatation and distortion in colon.

1. JEJUNAL CHANGES

1.1. Villous stunting

No change
Villus-height to crypt-depth (crypts of Lieberkühn) ratio is generally over 2 with slender smooth-surfaced villi.

Minimal (grade 1)
Villus to crypt ratio is 1–2. The villi are slightly decreased in height.

Mild (grade 2)
Villus to crypt ratio is 1–1.5. The villi are mildly decreased in height and the crypts are often elongated (see 1.3.).

Moderate (grade 3)
Villus to crypt ratio is <1–1.5. The villi are increased in width and some are fused. The crypts are often elongated.

Marked (grade 4)
Villus to crypt ratio is <1–1.5. The villi are generally fused. The crypts are often elongated.

1.2. Villous epithelial injury

Minimal injury
Epithelial cells show focally slight degenerative changes: increased eosinophilia and/or foamy cytoplasm. However, they palisade evenly and show little alteration in size or shape. No alteration in the number of goblet cells and intraepithelial lymphocytes.

Mild injury
Cells show modest degenerative changes; they are mildly swollen with foamy or vacuolar cytoplasm, or attenuated with increased eosinophilia. Subepithelial lamina propria may show edema and mildly increased numbers of lymphocytes. The number of goblet cells may be slightly decreased and the stem of the villi may contain flattened, basophilic regenerative cells.

Moderate injury
Epithelium exhibits desquamation and some apoptotic cells. Epithelial cells show at least focally marked degeneration being low columnar to cuboidal, intensely eosinophilic and often vacuolar. The number of goblet cells may be markedly decreased and the stem of the villi may contain flattened, basophilic regenerative cells. Subepithelial lamina propria may contain eosinophils, lymphocytes and single neutrophils.

Marked injury
Epithelial cells are markedly degenerated. The villi have widespread surface epithelium loss or ulceration while at the stem of the villi the cells are flattened and regenerating.

1.3. Epithelial injury in crypts, Paneth cell injury and Crypt hyperplasia, jejunum

Crypt epithelial injury and Paneth cell injury were associated, while crypt hyperplasia was generally negatively correlated with them but influenced assessment of villous stunting. Minimal epithelial injury is present only in colon samples.

Mild epithelial injury (grade 2)
Epithelial cells are mildly degenerated and show mild dysplastic changes: generally plemorphism and disorganization, and often large vesicular nuclei with pronounced nucleoli. The number of goblet cells is decreased and epithelium may contain single apoptotic cells.

Moderate epithelial injury (grade 3)
Crypts are often distorted and crypt epithelial cells are dysplastic and diffusely or multifocally degenerated. Epithelium exhibits considerable number of apoptotic/necrotic cells and single crypts may be necrotic.

Marked epithelial injury (grade 4)
Crypts are distorted and crypt epithelial cells are degenerated or necrotic and there is intraepithelial eosinophil and neutrophil infiltration. Some crypts are collapsed and indiscernible and there are some crypt abscesses with squamous basophilic epithelium.

Mild Paneth cell injury (grade 2)
The number of Paneth cells is mildly decreased and the cells show degenerative changes (increased vacuolation and coarseness of granules).

Moderate (Marked) Paneth cell injury (grade 3)
Paneth cells are degenerated and their number is moderately to markedly decreased (absent in two samples).

Mild crypt hyperplasia (grade 2)

Crypts are mildly dilated and distorted and crypt epithelial cells may be mildly dysplastic.

Moderate crypt hyperplasia (grade 3)
Crypts are moderately elongated and generally mildly dilated and/or distorted. Epithelial cells may be mildly dysplastic.

1.4. Lamina propria leucocytes (per leucocyte type), jejunum

No change
Lamina propria of the villi contains ≤5 *lymphocytes* per villus and between crypts they are present singly (1-3 on average); occasionally in clusters of few cells. There are ≤10 *eosinophils* per villus and among crypts they total up to 10 per 40x objective field (high power field; HPF), sometimes residing in small clusters. *Neutrophils* are absent.
N.B. Few plasma cells were present in any of the samples and are not mentioned separately.

Mild (per leucocyte type; grade 2)
Lamina propria of the villi contains 5–10 *lymphocytes* per villus. Crypts may be separated by up to 5 lymphocytes or plasma cells and they may infiltrate slightly below crypt bases. *Eosinophils* total up to 15 per average HPF, often residing in small clusters. *Neutrophil* infiltrates are lenient (5–10 per HPF); some cells may spread into epithelium.

Moderate (grade 3)
Lamina propria of the villi contains ≤ 20 *lymphocytes* per HPF. Crypts may be separated by up to 10 lymphocytes or plasma cells and they are present below crypt bases. *Eosinophils* total up to 25 per average HPF generally in clusters. *Neutrophil*s are present in moderate numbers, less than 15 per HPF.

Marked (grade 4)
Lymphocytes occupy over 50% of the villous lamina propria in a HPF. Crypts are separated by over 10 lymphocytes and they spread densely below crypt bases. *Eosinophils* dominate the tissue population of leucocytes and are not easily enumerated within a HPF. No sample showed marked *neutrophil* increase.

2. COLON

2.1. Surface epithelial injury, colon

Minimal injury
Epithelial cells show focally slight degenerative changes: increased eosinophilia and/or foamy cytoplasm. However, they palisade evenly and show little alteration in size or shape. No alteration in the number of goblet cells and intraepithelial lymphocytes.

Mild injury
Cells show modest degenerative changes; they are mildly swollen with foamy or vacuolar cytoplasm, or attenuated with increased eosinophilia. Subepithelial lamina propria may show edema and mildly increased numbers of lymphocytes.

Moderate injury
Epithelium exhibits desquamation and some apoptotic cells as well as occasional intraepithelial granulocytes. Epithelial cells show at least focally marked degeneration; they are low columnar to flattened, intensely eosinophilic and often vacuolar. Subepithelial lamina propria may contain eosinophils, lymphocytes and single neutrophils.

Marked injury
Present only in jejunal samples.

2.2. Crypt epithelial injury, Crypt distortion and dilatation, and Crypt hyperplasia in colon

Crypt epithelial injury, and crypt distortion and dilatation were associated. Colon samples showed only mild crypt hyperplasia.

Minimal epithelial injury
Epithelial cells are mildly degenerated and the number of goblet cells is decreased.

Mild epithelial injury
Epithelial cells are mildly degenerated and show mild dysplastic changes. The number of goblet cells is decreased and epithelium may contain single apoptotic cells.

Moderate epithelial injury
Apical-mid parts of some crypts are dilated and lined by flattened (even squamous), basophilic epithelium with occasional apoptotic cells. Dilatations contain mucus and epithelial cell remnants.

Marked epithelial injury
Most crypts have moderately increased diameter with prominent lumens. They contain mucus and epithelial cell remnants and are lined by flattened epithelium (see Moderate injury). In colon marked injury was present in only one sample.

Crypt distortion and dilatation is subjectively graded as minimal (1), mild (2) or moderate (3) based on crypt tortuousness (distortion), and lumen diameter and prominence. No marked distortion and dilatation existed.

Crypt hyperplasia was present as a mild (2) change in colon: Crypts are mildly elongated and distorted, and have generally mildly increased diameter. Their epithelium is basophilic and shows often mildly increased numbers of mitoses.

2.3. Crypt loss in colon

Crypt loss (atrophy) was graded based on loss of the crypts and increase of stromal immature connective tissue. No marked crypt loss appeared and moderate destruction was present only in one animal.

No change
Narrow band of stroma separates uniformly distributed crypts.

Minimal crypt loss
Crypts are focally separated by widened band of stroma and immature connective tissue and there is a definite loss of at least one crypt.

Mild crypt loss
Crypts are separated by widened band of stroma and immature connective tissue and there is a localized loss of more than one crypt.

Moderate crypt loss
Crypts are diffusely separated by widened band of stroma and immature connective tissue. Loss of crypts is accompanied by marked epithelial injury in the surviving crypts.

2.4. Lamina propria leucocytes (per leucocyte type), colon

No change
Less than 5 *lymphocytes* are present between crypts occasionally in small clusters. There are 5–10 *eosinophils* per HPF. *Neutrophils* are absent (single cells).
Few plasma cells were present in any of the samples and are not mentioned separately. No sample showed increase of *neutrophils*.

Mild (per leucocyte type; grade 2)
Crypts may be separated by up to 10 lymphocytes which infiltrate slightly below crypt bases. *Eosinophils* total up to 15 per average HPF, often residing in small clusters.

Moderate (grade 3)
Eosinophils total up to 25 per average HPF, generally in clusters. Present in one sample.

Marked (grade 4)
Present only in jejunal samples.
